# Prescription of Antibiotics to Treat Gonorrhoea in General Practice in Flanders 2009–2013: A Registry-Based Retrospective Cohort Study

**DOI:** 10.1155/2017/1860542

**Published:** 2017-07-31

**Authors:** Christoph Schweikardt, Geert Goderis, Steven Elli, Yves Coppieters

**Affiliations:** ^1^Université libre de Bruxelles (ULB), School of Public Health, Epidemiology, Biostatistics, and Clinical Research (Research Center 2) and Health Policies and Systems-International Health (Research Center 3), Campus Erasme, Bâtiment A, CP 594, route de Lennik 808, 1070 Brussels, Belgium; ^2^Catholic University of Leuven, Academic Center for General Practice, Kapucijnenvoer 33, Blok J, Bus 7001, 3000 Leuven, Belgium

## Abstract

**Background:**

General practitioners (GPs) as a group have been identified as playing an important role in gonorrhoea management in Flanders. Belgian guidelines recommended ceftriaxone or alternatively spectinomycin from 2008 onwards and azithromycin combination therapy since 2012.

**Objectives:**

This study investigates to which extent contemporary gonorrhoea treatment guidelines were followed.

**Methods:**

A retrospective cohort study (2009–2013) of antibiotic prescriptions for gonorrhoea cases registered in the Flemish Intego general practice database was carried out. The database is based on electronic health record routine registration by over 90 GPs using the software programme Medidoc.

**Results:**

Ninety-one gonorrhoea cases with ten chlamydia and one genital trichomonas coinfections in 90 patients were registered between 2009 and 2013. The proportion of cases with ceftriaxone and/or spectinomycin prescriptions rose from 13% (two of 15 cases) in 2009 to 56% (nine of 16 cases) in 2013. Combination therapy of ceftriaxone and/or spectinomycin together with azithromycin rose from 0 of 15 cases (0%) in 2009 to 7 of 16 cases (44%) in 2013.

**Conclusion:**

Although numbers are small, the results suggest that gonorrhoea therapy guideline adherence improved between 2009 and 2013.

## 1. Introduction

As in other parts of Europe [1, pp. 19-20], the number of newly diagnosed gonorrhoea cases has been increasing in Flanders since 2009 [2, p. 26]. Figures from the Flemish Agency for Care and Health [3] show an increase from 622 gonorrhoea cases in 2009 to 1162 cases in 2013 [4]. That year, the Flemish Minister of Health confirmed the need to reinforce measures against sexually transmitted infections (STIs) [5].

No comprehensive surveillance system for gonorrhoea exists in Belgium. Instead, the following complementary surveillance systems exist: first, the Belgian Network of Sentinel Laboratories for Microbiology [6, p. 15] covers about 50% of diagnostic activity [2, p. 6]; the Belgian Network of Sentinel STI Clinics/Clinicians in cooperation with the Belgian Network of Sentinel General Practices records STI cases including risk determinants and behaviour [6, pp. 16–18]; and finally, gonorrhoea is subject to mandatory notification to the Agency for Care and Health in Flanders [7] and to the respective health authorities in the Brussels-Capital Region [8, p. 11]. The case definition for mandatory notification of gonorrhoea [9, p. 49] subsumes probable cases (clinically suspected, after recent sexual contact with confirmed case) and confirmed cases (clinically compatible with laboratory confirmation, namely culture of* N. gonorrhoeae*, antigen test or PCR).

The World Health Organization recommends avoidance of an antibiotic when a 5% resistance is reached [10, p. 14] and points out that multidrug resistance of gonorrhoea strains against antibiotics constitutes a growing public health threat [11, 12]. In 2009, the Belgian National Reference Laboratory for Neisseria gonorrhoeae found Neisseria gonorrhoeae resistance against penicillin, tetracycline, and ciprofloxacin in 35.5%, 48.0%, and 57.5% of the strains, respectively [13, p. 39]. “Resistance against azithromycin decreased from 3.1% in 2007 to 1.6% in 2008. All strains were susceptible to ceftriaxone and spectinomycin” [13, p. 39].

Changing resistance patterns of gonococci led to the adaptation of respective national guidelines. From 2008 onwards, the Belgian Antibiotic Policy Coordination Committee (BAPCOC) recommended the cephalosporin ceftriaxone as first choice or the aminoglycoside spectinomycin for eradication of gonococci instead of a quinolone (ofloxacin, ciprofloxacin, and levofloxacin) ([14, p. 53, p. 58]; [15, p. 7, p. 62]; [16, p. 60]). In 2008, BAPCOC recommended azithromycin or doxycycline for etiological treatment of chlamydia [15, p. 62] and in combination with ceftriaxone for empirical treatment of urethritis [15, p. 62]. In 2012, BAPCOC gave azithromycin a more prominent place for eradication of gonococci as part of a combination therapy together with ceftriaxone or spectinomycin [16, p. 60].

BAPCOC did not rule out entirely other antibiotic classes: quinolones remained for gonococcal urethritis if sensitivity was proven ([15, p. 62]; [16, p. 60]). In 2008, BAPCOC continued to recommend ofloxacin and levofloxacin as empiric treatment for prostatitis and orchiepididymitis, with ceftriaxone to be added if gonococci were suspected [15, p. 54-55]. In 2012, the first choice for prostatitis in case of suspected gonococci became ceftriaxone or spectinomycin with azithromycin [16, p. 52] and ceftriaxone or spectinomycin with doxycycline for orchiepididymitis in case of a suspected STI [16, p. 53]. BAPCOC recommended amoxicillin with clavulanic acid or cotrimoxazole for prostatitis and amoxicillin with clavulanic acid or cefuroxime axetil for orchiepididymitis as alternative or second choice ([15, p. 54-55]; [16, p. 52-53]).

The Flemish gonorrhoea treatment guidelines (October 2009) [17] followed BAPCOC and ceftriaxone or spectinomycin as treatment of choice for gonorrhoea eradication and to combine azithromycin for chlamydia [17, p. 215]. In May 2013, the recommendation of combination therapy of ceftriaxone or spectinomycin with azithromycin for gonorrhoea followed [18].

Laisnez and colleagues found in 2010 that the general practitioner (GP) in the provinces of East and West Flanders was the treating physician in 79.1% of gonorrhoea cases [19, p. 5] and that 55.7% of GPs adhered to current guidelines, defined as treatment with ceftriaxone and/or spectinomycin, whereas GPs prescribed ciprofloxacin in 28.7% of cases [19, p. 5]. Aside from this study and their follow-up study for 2012–2014 [20], knowledge about antibiotic gonorrhoea treatment choices in general practice in Flanders is limited. Therefore, this study investigates (a) which antibiotics for gonorrhoea treatment were registered in the Intego general practice database for 2009–2013 and (b) to which extent Belgian/Flemish gonorrhoea treatment guidelines were followed.

## 2. Methods

### 2.1. Setting

The Flemish Intego network (for details see Truyers et al. [21], Truyers et al. [22], and Vaes et al. [23]) is “the only operational computerized morbidity registration network in Belgium based on general practice data” [21, p. 1]. Over 90 Intego GPs (see Table 1), who are good coders, collect data on about 2% “of the Flemish population representative in terms of age and sex” [21, p. 1]. “Intego procedures were approved by the ethical review board of the Medical School of the Catholic University of Leuven (N° ML 1723) and by the Belgian Privacy Commission (number SCSZG/13/079)” [23, p. 10].

All Intego GPs work with the proprietary software programme Medidoc®. GPs routinely register all new diagnoses and new drug prescriptions which are collected together with patient information from GPs' personal computers and entered into a central database [23, p. 2]. There is no interference with the daily work of the GP [22, p. 16]. GPs are requested to encode clinical labels (keywords) offered by the software programme. The classification system is proprietary: to each clinical label (keyword) Medidoc assigns a programme-specific internal Medidoc code and a “diagnostic group” code, for example, for gonorrhoea, syphilis, trichomonas, and chlamydia. Furthermore, it links new diagnoses to the International Classification of Primary Care (ICPC-2) and the International Statistical Classification of Diseases and Related Health Problems, 10th Revision (ICD-10) [23, p. 2].

### 2.2. Study Design

In order to investigate antibiotic treatment choices after the publication of the 2008 BAPCOC recommendations, this retrospective cohort study used Intego data of a 5-year period from 1 January 2009 to 31 December 2013. The population of interest was constituted by the practice population, calculated from the yearly contact group plus the group which did not visit their general practitioner in a given period (method: see [24]).

We selected the relevant Medidoc codes for gonorrhoea, urogenital* Chlamydia trachomatis* infections, syphilis, and genital trichomoniasis together with the diagnostic group variable representing these four STIs (see Supplementary File, Annex 1, in Supplementary Material available online at https://doi.org/10.1155/2017/1860542). Observations with these codes were extracted together with the patient number and the beginning date of the respective diagnosis. The number of gonorrhoea infections per year was counted as episodes (cases), consisting of one or more patient consultations for the same medical diagnosis.

In the scientific literature, there are various approaches to define the interval distinguishing a new from an ongoing genital chlamydia or gonorrhoea episode (or STI episodes in general) in a database. Regarding the Belgian Network of Sentinel Laboratories for Microbiology, an interval of more than 90 days between two positive chlamydia or gonorrhoea results was defined before a second positive result was counted as a new episode [2, p. 6]. In their article on genital* Chlamydia trachomatis* infections, Hughes and colleagues stated that “events occurring within 30 days of each other formed part of the same test, diagnosis, treatment, or referral episode” [25, p. 311]. Subsequently, “all events occurring within 30 days of each other were grouped as a single episode,” and an alternative episode definition of 60 days “was found to have negligible impact on incidence estimates” [25, p. 311]. In their study on STIs in sexual health clinics in England, Mohammed and colleagues counted only 1 diagnosis of each STI within a 6-week period [26, p. 88]. For their “Comparison of STI-Related Consultations among Ethnic Groups in the Netherlands,” Woestenberg and colleagues [27] used the application EPICON “that groups consultations with similar ICPC-codes occurring less than two months apart” ([27, p. 2]; [28]). Following the example of Suijkerbuijk and colleagues on* Chlamydia trachomatis* infections diagnosed by GPs in the Netherlands [29, p. 3], we counted a second episode with the same diagnosis for the same patient as a new case only after an interval of at least two months after the beginning date of the first diagnosis. We defined the two month-interval as 62 days.

Observations of chlamydia, syphilis, and genital trichomonas were kept only if they concerned gonorrhoea coinfections. We compared two definitions of “coinfection”: we took the “date of beginning of diagnosis” of the gonorrhoea infection as point of origin. According to Definition 1, a registration of a* Chlamydia trachomatis*, syphilis, or genital trichomonas infection was counted as coinfection if it was registered with its “day of beginning of diagnosis” during an interval of up to seven days before/after the gonorrhoea “date of beginning of diagnosis.” According to Definition 2, the interval of 7 days was replaced by an interval of 14 days before/after the “day of beginning of diagnosis” of the gonorrhoea infection. Applying the 14-day period led to the detection of one further chlamydia infection. The patient had been prescribed erythromycin at the gonorrhoea infection registration date, and 12 days later doxycycline, when chlamydia was registered. We decided that the case was congruent with a coinfection and kept the latter definition.

For one patient, two gonorrhoea cases were recorded, but only for the second case antibiotic therapy was recorded. Subsequently, the patient number for the first case was changed in order to obtain a set of unique patient numbers in the first file. Based on the patient number, the file was merged with a second one containing year of birth and sex. For the patient whose patient number had been changed, year of birth and sex were added manually.

Then we selected ATC group codes (Anatomical Therapeutic Chemical Classification System, for details, see [30]) of the anti-infectives (see Supplementary File, Annex 2) and extracted matching observations together with the patient number, the name of the prescribed medication, and the prescription date. Subsequently, the data file was merged with the previous one based on the patient number.

Dermatological and sensory antibiotics (with ATC codes beginning with D and S) were not included in the analysis. If the same antibiotic (defined as antibiotic with the same ATC code) was given more than once in the period fourteen days before until fourteen days after the beginning date of gonorrhoea diagnosis, then this was counted as therapy with one antibiotic. Thus, we obtained the ATP codes prescribed in a close temporal context of each recorded gonorrhoea case.

We had to determine the period of time between registration date of the beginning of gonorrhoea diagnosis and registration date of the antibiotic judged as sufficient to cover empiric therapy, based on symptoms suspecting venereal disease without having a PCR or culture result, as well as possibly changing therapeutic choices after obtaining the positive laboratory result after the patient consultation.

Since this was the first investigation into gonorrhoea therapy based on Intego data, we compared two time spans: the first covered antibiotics registered within one week before/after the beginning date of the gonorrhoea diagnosis and the second two weeks.

By enlarging the period to 14 days before/after the gonorrhoea registration date, 8 additional antibiotics (excluding the same antibiotic prescribed within 7 days and again within the 14-day period) were found: azithromycin twice, doxycycline twice, ciprofloxacin twice, moxifloxacin once, and metronidazole once. One additional antibiotic was found in six cases and two in one case. Since it concerned antibiotics expected for the therapy of sexually transmitted infections, we kept the larger interval.

We grouped the cases according to number and combination of ATP codes prescribed. Combination therapy was defined as prescription of two or more systemic antibiotics with different ATC codes within the period fourteen days before until fourteen days after the beginning date of a gonorrhoea diagnosis.

Due to the protection of patient privacy, we had obtained the year of birth, but not the birth date of the respective patients. In order to keep the error of the age estimation within half a year or less, we assigned July 1 of the birth year as fictitious birthday and estimated the age as the difference of the recorded beginning date of the gonorrhoea diagnosis and the fictitious birthday. We chose four age classes, namely, 0–14 years, 15–24 years, 25–44 years, and 45+ years. Descriptive analysis was performed with STATA 12.0 (StataCorp, Texas) and Excel 2010.

## 3. Results

### 3.1. Annual Frequency of Gonorrhoea Cases per GP

During the observation period 2009–2013, between 98 (minimum) and 116 GPs (maximum) participated annually, observing an annual patient population fluctuating around an order of magnitude of 150,000 (see Table 1). In total, 91 gonorrhoea cases were registered, including two cases referring to the same patient, one in November 2012 and one in April 2013. No observation was excluded for being registered a second time in less than 2 months. In 11 cases, bacterial/protozoal gonorrhoea coinfections were registered, namely, chlamydia ten times and genital trichomonas once. No syphilis coinfection was recorded.

### 3.2. Age and Sex Distribution

The 91 gonorrhoea cases included 76 males and 15 female cases, thus a ratio of males to females of 5/1. There were no cases under the age of 15 years. 43 of 76 male cases (57%) belonged to the age class 25–44 years, whereas the female cases were quite evenly spread among the age classes 15–24 years (6 cases, 40%), 25–44 years (4 cases, 27%), and 45+ years (5 cases, 33%). The age and sex distribution is shown in Table 2 (see also [31]).

### 3.3. Prescribed Antibiotics

In 78 of the 91 cases (86%), systemic antibiotic therapy was recorded. Antibiotics with 16 different ATC codes were prescribed.

In 35 cases (38%), one antibiotic was recorded, most often a quinolone (12 cases), a penicillin derivative (amoxicillin with/without enzyme inhibitor clavulanic acid, flucloxacillin, seven cases), or ceftriaxone (six cases). In 33 cases (36%), two different antibiotics were recorded in 18 different ATC code combinations, most often ceftriaxone with azithromycin (nine cases). In ten cases (11%), three or more different antibiotics were prescribed. In general, combination therapies which included penicillin derivatives played a minor role with five cases, including one combination therapy with spectinomycin, one with azithromycin, and none with ceftriaxone. An overview is given in Table 3.

Cases with coinfections showed a wide variety of therapeutic choices. For three of the ten gonorrhoea-chlamydia coinfections, only one antibiotic (ciprofloxacin in 2010, doxycycline in 2011, and azithromycin in 2012, resp.) was recorded. In total, six of the ten gonorrhoea-chlamydia coinfections received azithromycin prescriptions. Four of the gonorrhoea-chlamydia coinfections were treated with ceftriaxone and/or spectinomycin together with azithromycin, all from 2011 onwards. The single gonorrhoea/trichomonas coinfection in 2013 was treated with azithromycin and metronidazole.

Contrary to male gonorrhoea cases which showed a wide spectrum of therapeutic choices, all 11 female cases with registered anti-infective therapy were treated with either amoxicillin alone (3 cases), ceftriaxone alone (4 cases), a combination of ceftriaxone and azithromycin with or without a third anti-infective (3 cases), or azithromycin with metronidazole (1 case, gonorrhoea-trichomonas coinfection). No spectinomycin, no quinolone, and no doxycycline were registered in female cases. No clustering of age classes regarding antibiotic prescriptions could be detected.

### 3.4. Prescription of Quinolones

The number of cases with quinolone therapy decreased continuously from 11 of 15 cases (73%) in 2009 to two of 16 cases (13%) in 2013. Combination therapy of a quinolone with ceftriaxone and/or spectinomycin was recorded for one case each year except in 2011 (three cases). In eight cases, the therapy included quinolone(s) and doxycycline (five cases in 2009, one in 2010, and two in 2012).

### 3.5. Prescription of Cephalosporins and/or Spectinomycin and Their Combination with Azithromycin

The proportion of ceftriaxone and/or spectinomycin prescriptions increased continuously throughout the observation period: the number of cases with prescription of ceftriaxone rose from 0 of 15 cases (0%) in 2009 to nine of 16 cases (56%) in 2013, while spectinomycin was prescribed twice in 2009, reached a peak of four cases in 2012, and was not recorded in 2013. Thus, the annual number of cases with prescription of ceftriaxone and/or spectinomycin rose from two of 15 (13%) in 2009 to nine of 16 cases (56%) in 2013. Cases with combination therapy of ceftriaxone and/or spectinomycin with azithromycin rose from 0 of 15 cases (0%) in 2009 to seven of 16 cases (44%) in 2013 (see Table 4).

Cephalosporins other than ceftriaxone were prescribed three times, cefuroxime with ciprofloxacin once in 2009, cefotaxime with doxycycline and azithromycin once in 2012, and cefuroxime alone once in 2013, bringing the number of cases with a cephalosporin prescription in 2013 up to ten of 16 cases (63%).

## 4. Discussion

### 4.1. Defining a Proxy for Gonorrhoea Therapy Guideline Adherence

This study gives new insight into antibiotic prescriptions for gonorrhoea therapy recorded in general medicine in Flanders, notably the variety of treatment choices involved. They are based on routine registration of physicians and thus without the bias of GPs paying special attention to either gonorrhoea or STI therapy.

Given the fact that ceftriaxone and spectinomycin were recommended throughout the observation period, that azithromycin was recommended formally since 2012/2013, and that it takes time to spread the new version of a guideline, the best proxy for gonorrhoea guideline adherence 2009–2012 seems to be the prescription of ceftriaxone and/or spectinomycin, with 2013 being a transition year, and from 2014 onward the combination therapy of ceftriaxone and/or spectinomycin together with azithromycin.

### 4.2. Gonorrhoea Cases without Recorded Treatment

For one-seventh of gonorrhoea cases (13 of 91), no treatment is recorded. Due to privacy protection procedures, the research team at the Academic Center of General Practice of KU Leuven is blinded to the GPs' origin. It cannot be ruled out that the patient was diagnosed but not treated (e.g., was not treated empirically and did not show up any more after the diagnosis arrived). However, it is more likely that the patient was treated but the prescription of the antibiotic was not registered. One possibility is that the GP did not register the prescribed antibiotic within the period of 14 days before/after the beginning date of the gonorrhoea diagnosis. Another possibility is that the patient was treated by the specialist or another health care provider and that the GP, notified by the health care provider, registered the diagnosis but not the treatment.

### 4.3. Proportion of Female Cases and Prescriptions for Female Gonorrhoea Patients

The proportion of male (83.5%) and female (16.5%) gonorrhoea cases in this study is comparable with the ones found by Laisnez and colleagues in 2010 (81.7% men, 18.3% women) and 2012–2014 (79.0% men, 21.0% women) [20, p. 19].

The absence of quinolones and doxycycline in female gonorrhoea treatment may be due to several factors: (1) two-thirds of cases (10 of 15) occurred in 2012 and 2013, with ceftriaxone having been recommended as treatment of choice for several years; (2) ceftriaxone, azithromycin, and amoxicillin are considered safe/probably safe drugs in pregnancy so that they can be prescribed easily even if pregnancy is not ruled out, whereas quinolones and doxycycline are less recommended during pregnancy [32, 33]; (3) possibly, there is a clustering of few Intego GPs consulted by these women leading to little variation in treatment choices.

### 4.4. Guideline Adherence according to the Intego Database

Although the figures are small, the data suggest that gonorrhoea therapy guideline adherence improved between 2009 and 2013. After the Belgian BAPCOC guideline recommendations of 2008, it took five years, until 2013, before more than half of Intego-registered gonorrhoea cases were treated according to these guidelines, defined as prescription of ceftriaxone and/or spectinomycin.

We do not know whether a combination therapy of ceftriaxone or spectinomycin together with azithromycin after publication of the new guidelines in 2012/2013 was due to knowledge of the new guidelines or a therapeutic choice which was based on previous recommendations and aiming at covering a (potential) chlamydia infection as well. We interpret the choice of ceftriaxone or spectinomycin, both intramuscular drugs, instead of an oral antibiotic as a strong hint that at least the recommendations of 2008 were known.

As expected, it took time for the 2008 guideline to be implemented in general practice. For 2010, recorded gonorrhoea therapy guideline adherence of Intego GPs was only 18%. Even if we assume that Intego cases without recorded therapy are referred or reported cases, for which the GP did not record the therapy, guideline adherence of Intego GPs in 2010 was only 24% (4 of 17 cases). This figure is much lower than the one for GPs in the study of Laisnez and colleagues for 2010 in the provinces East and West Flanders (55.7%). Sending the treatment guidelines to the treating physician might have had a positive influence on guideline adherence. The follow-up study of Laisnez and colleagues showed 65.3% guideline adherence (defined as ceftriaxone and/or spectinomycin) for 2012 and 53.0% new guideline adherence (defined as ceftriaxone and/or spectinomycin together with azithromycin) in 2013-2014 for all cases with known treatment [20, p. 21] (not only GPs; separate figures for GPs were not published, but the GP was the treating physician in 79.8% of cases [20, p. 19]).

These results are slightly higher than Intego figures for 2012 and about equal in 2013 if only cases with recorded therapy are taken into account: 53% (9 of 17) of cases in 2012 (ceftriaxone and/or spectinomycin) and 54% (7 of 13) of cases in 2013 (ceftriaxone and/or spectinomycin together with azithromycin).

We assume that Intego figures of Table 4 on the proportion of cases treated according to contemporary guidelines represent the lower limit of cases treated according to contemporary guidelines by Intego GPs. Given that gonorrhoea is not a frequently encountered disease for the GP, gonorrhoea therapy guideline adherence in 43% of cases in 2012 (prescription of ceftriaxone and/or spectinomycin) and in 44% of cases in 2013 (combination therapy with azithromycin, if applying the new guidelines of 2012/2013) already constitutes a considerable achievement. In the same year, a much more common condition, the proportion of adult diabetics, aged 50+, under oral antidiabetics only, with appropriate follow-up in Flanders ranged with 43.5% in the same order of magnitude [34, p. 27].

Moderate guideline adherence has also been found in other European settings. In general practice in England, less than half of GP-treated episodes received a recommended gonorrhoea regimen over the study period 2000–2011 [35, p. 6-7]. Falchi and colleagues found in 2008 that slightly more than 40% of responding sentinel GPs in France fully completing the vignette prescribed two recommended antibiotics or one recommended antibiotic against gonorrhoea [36, p. 3]. Since only 35% of GPs responded to a fictitious case of chlamydia-gonorrhoea coinfection instead of actual treatment choices in practice, the proportion of French sentinel GPs treating with one or more recommended antibiotics may or may not have been substantially higher than in the Intego network one year later.

Overall, Intego data confirm the dilemma pointed out by Laisnez et al. ([19, p. 6]) that GPs in Flanders rarely see gonorrhoea cases in their routine practice, but that they as a group are important for the fight against gonorrhoea. Furthermore, Vandenbruaene and Crucitti have shown that the issue is not only knowing the latest guidelines, but also the variety of them and their updates, since one of the other guidelines recommended ciprofloxacin still in 2013 [37, slides 55–58]. According to Boffin and colleagues, “time has come for a general practice guideline on STI management, including recommendations on HIV testing and discussing of partner notification” [38, p. 7]. Preparation is under way: the Belgian Health Care Knowledge Centre (KCE) plans a “Guideline on Sexually Transmitted Diseases” study [39].

### 4.5. Low Number of Overall Gonorrhoea Cases

More than 90 Intego GPs reported a total of 91 cases in 5 years, as a mean less than 1 case per GP in 5 years. Elsewhere, we argued that there seems to be not much underreporting of gonorrhoea cases to mandatory notification in Flanders and that Intego figures suggest GP involvement in most gonorrhoea cases [31]. Another issue is that many infections may not have been detected. According to Domus Medica, the association of Flemish GPs which represents their interests and supports them scientifically [40], few STIs are diagnosed by GPs in comparison to other professions, although the GP quite often is the first contact person [41]. In this context, a STI teaching module for GPs has been developed, serving as a guide to improve quality of care [42, 43]. Researchers from the Belgian Scientific Institute of Public Health have also recommended training GPs in STI consulting and in opportunistic screening with risk factor awareness [44].

### 4.6. Limitations of the Study

The reasoning that there is a link between gonorrhoea diagnosis and antibiotics prescribed is based on the proximity of the registration dates without an established link in the data. However, in 2008 and 2012, BAPCOC recommended ceftriaxone for few other indications in ambulatory care other than genitourinary and sexually transmitted infections which could be caused by* N. gonorrhoeae* (syphilis in case of penicillin allergy ([15, p. 64]; [16, p. 62]) and carriers/prophylaxis of meningococci ([15, p. 74]; [16, pp. 70-71])), and spectinomycin was recommended only for gonococci. Furthermore, they are intramuscular drugs and therefore less comfortable to administer than oral treatment. Azithromycin is more broadly indicated, including respiratory, urogenital, dermal, and other bacterial infections ([15, 16, 45]). We do not know whether or not a registered antibiotic might have been prescribed for another concomitant infection at the same time.

In the Intego network, GP judgement is pivotal for registering a gonorrhoea clinical label leading to a gonorrhoea diagnostic group Medidoc code. Underregistration cannot be excluded, although Intego takes pains to recruit proven good coders. We do not know whether the GP based his/her judgement on laboratory and/or microscopy results, as recommended in the guidelines, or whether it concerned empiric or partner therapy. In case of prescription according to the guidelines, they may even have been sent to the GP by the Flemish health authorities in the course of mandatory notification. Some patients with a negative test result may have been treated presumptively, and these were not included in our analyses. Correct dosages or the number of days to take the medication could not be controlled.

The variable “date of beginning of diagnosis” may refer to the date of first patient symptoms, the date of patient consultation, or the date when the laboratory confirmation of gonorrhoea arrived, so that it cannot be established with certainty whether the antibiotic was prescribed before knowledge about the etiology was attained.

At present, the generalisability of the findings to GPs of all Flanders is not known. Intego is representative of the Flemish population, but we do not know whether gonorrhoea treatment choices (as well as STI detection strategies and cooperation with specialists) of Intego GPs are representative of all GPs in Flanders. Truyers and her colleagues at the Academic Center of General Practice at the Catholic University of Leuven assume that Intego GPs probably only differ from their colleagues in their handling of medical software but not in their medical interventions or the composition of their patients [22, p. 15]. However, given the fact that Intego GPs are good coders and accept that their data are extracted, we can assume as well that they are more than average devoted to their professional duties and more dedicated to implement new guidelines into their daily routine. The percentage of gonorrhoea treatment choices not according to guidelines in Flanders may therefore rather be larger than smaller compared with the one registered in Intego.

### 4.7. Opportunities for Future Research

The Intego database itself will be developed further. For the moment, Intego is planning a change in the registration software system in order to take advantage of the modern possibilities in electronic data-capture and transfer, enabled by the national eHealth Roadmap [46]. This step will open new ways and opportunities for surveillance and monitoring of diseases.

Furthermore, the extraction of antibiotic prescriptions for gonorrhoea could be repeated annually, as soon as the data from the respective year become available. Finally, Flemish GPs with electronic health records other than Medidoc could be requested to send their STI data, if this is technically feasible, in order to examine the generalisability of Intego to all Flanders.

## 5. Conclusion

Given the global threat of antimicrobial resistance and substantial GP involvement in gonorrhoea control in Flanders, monitoring GPs' therapeutic choices including adherence to national treatment guidelines remains important, especially if monitoring is possible from routine registration. Data from the Intego database give insight into the variation of gonorrhoea treatment options registered by GPs in Flanders during the observation period 2009–2013. Although limited by small numbers, they suggest a gradual increase of antibiotic prescriptions according to gonorrhoea guidelines. The Intego database offers a tool in place for annual monitoring of general practitioners' therapeutic choices to treat gonorrhoea. Further research is necessary in order to determine whether the Intego database offers a suitable proxy for monitoring Intego GP gonorrhoea therapy guideline adherence, with indicators ceftriaxone/spectinomycin and azithromycin included in the prescription within 14 days before/after the registered beginning date of gonorrhoea.

## Supplementary Material

The Supplementary Material contains (1) the selected Medidoc codes, the associated diagnostic group code, the Medidoc clinical label in Dutch and its English equivalent; (2) ATC codes for the selection of antiinfectives from the Intego database.

## Figures and Tables

**Table 1:** Number of annually participating general practitioners (GPs), patient population, annual number of gonorrhoea cases per participating GP, and annual number of bacterial/protozoal coinfections, Intego database, 2009–2013.

Year	2009	2010	2011	2012	2013	Total
Number of participating GPs	111	116	109	105	98	
Patient population	159698	156068	166740	137796	141631	
Number of gonorrhoea cases	15	22	17	21	16	91

Coinfections of chlamydia	1	2	4	3	0	10
Coinfections of genital trichomonas	0	0	0	0	1	1
Coinfections of syphilis	0	0	0	0	0	0

**Table 2:** Age class and sex distribution of gonorrhoea cases (*n* = 91), Intego database, 2009–2013.

Age class	Male	% of males	Female	% of females	Total
1 (<15 years)	0	0%	0	0%	0
2 (15–24 years)	17	22%	6	40%	23
3 (25–44 years)	43	57%	4	27%	47
4 (45+ years)	16	21%	5	33%	21

Total	76	100%	15	100%	91

**Table:** (a) Male cases (*n* = 76)

	ATC code of antibiotic 1/antibiotic substance 1	ATC code of antibiotic 2/antibiotic substance 2	ATC code of antibiotic 3/antibiotic substance 3	ATC code of antibiotic 4/antibiotic substance 4	2009	2010	2011	2012	2013	Total
Total number of gonorrhoea cases recorded					**14 ** **(1)**	**20 ** **(2)**	**15 ** **(4)**	**16 ** **(2)**	**11**	**76 ** **(9)**

No antibiotic recorded					**1** **[2]**	**4** **[2**,**3,3,3]**	**0**	**2** **[2, 3]**	**2** **[3, 3]**	**9**

One antibiotic recorded	J01DD04 Ceftriaxone				0	0	0	2 [2, 3]	0	**2**
	J01DC02 Cefuroxime				0	0	0	0	1 [3]	**1**
	J01XX04 Spectinomycin				0	0	0	1 [2]	0	**1**
	J01FA10 Azithromycin				1 [3]	1 [3]	0	1 (1) [3c]	1 [3]	**4 (1)**
	J01AA02 Doxycycline				0	2 [3, 3]	1 (1) [3c]	1 [4]	0	**4 (1)**
	J01CA04 Amoxicillin J01CF05 Flucloxacillin J01CR02 Amoxicillin and enzyme inhibitor				0	2 [3, 4]	1 [4]	1 [3]	0	**4**
	J01MA01 Ofloxacin J01MA02 Ciprofloxacin J01MA12 Levofloxacin J01MA14 Moxifloxacin				3 [2, 2, 3]	5 (1) [3, 3, 3, 3, 4c]	3 [2, 3, 3]	1 [4]	0	**12 (1)**

One antibiotic, number of cases					**4**	**10 ** **(1)**	**5 (1)**	**7 (1)**	**2**	**28 ** **(3)**

Two different antibiotics recorded	J01DD04 Ceftriaxone	J01FA10 Azithromycin			0	0	2 (1) [2c, 3]	1 (1) [3c]	5 [2, 3, 3, 3, 3]	**8 (2)**
	J01DD04 Ceftriaxone	J01AA02 Doxycycline			0	1 [4]	0	0	0	**1**
	J01DD04 Ceftriaxone	J01MA02 Ciprofloxacin			0	0	2 [3, 3]	0	1 [4]	**3**
	J01DC02 Cefuroxime	J01MA02 Ciprofloxacin			1 [4]	0	0	0	0	**1**
	J01XX04 Spectinomycin	J01FA10 Azithromycin			0	0	0	1 [2]	0	**1**
	J01XX04 Spectinomycin	J01MA02 Ciprofloxacin			0	0	1 [3]	0	0	**1**
	J01XX04 Spectinomycin	J01AA02 Doxycycline			0	1 [4]	0	0	0	**1**
	J01XX04 Spectinomycin	J01CA04 Amoxicillin			1 [4]	0	0	0	0	**1**
	J01FA01Erythromycin	J01AA02Doxycycline			0	1 (1) [3c]	0	0	0	**1 (1)**
	J01FA10 Azithromycin	J01AA02 Doxycycline			0	0	0	1 [2]	0	**1**
	J01FA10 Azithromycin	J01CR02 Amoxicillin and enzyme inhibitor			0	0	1 [3]	0	0	**1**
	J01FA10 Azithromycin	J01MA01 Ofloxacin			0	0	0	0	1 [4]	**1**
	J01FA10 Azithromycin	J01MA02 Ciprofloxacin			0	0	2 (1) [2c, 3]	0	0	**2 (1)**
	J01MA02 Ciprofloxacin	J01CA04 Amoxicillin			1[3]	0	0	0	0	**1**
	J01MA02 Ciprofloxacin	J01AA02 Doxycycline			4 [2, 3, 3, 4]	0	0	1 [3]	0	**5**
	J01MA12 Levofloxacin	J01CA04 Amoxicillin			0	1 [4]	0	0	0	**1**
	P01AB01 Metronidazole	J01AA02 Doxycycline			0	0	1 [3]	0	0	**1**

Two antibiotics, number of cases					**7**	**4 (1)**	**9 (2)**	**4 (1)**	**7**	**31 ** **(4)**

Three different antibiotics recorded	J01DD04 Ceftriaxone	J01XX04 Spectinomycin	J01FA10 Azithromycin		0	0	0	1 [4]	0	**1**
	J01DD04 Ceftriaxone	J01FA10 Azithromycin	J01MA02 Ciprofloxacin		0	1 [3]	0	0	0	**1**
	J01DD01 Cefotaxime	J01FA10 Azithromycin	J01AA02 Doxycycline		0	0	0	1 [2]	0	**1**
	J01XX04 Spectinomycin	J01MA12 Levofloxacin	P01AB01 Metronidazole		1 (1) [3c]	0	0	0	0	**1 (1)**
	J01MA12 Levofloxacin	J01AA02 Doxycycline	J01CA04 Amoxicillin		0	1 [2]	0	0	0	**1**

Three antibiotics, annual number of cases					**1 (1)**	**2**	**0**	**2**	**0**	**5 (1)**

Four different antibiotics recorded	J01DD04 Ceftriaxone	J01FA10 Azithromycin	J01XX04 Spectinomycin	J01XE01 Nitrofurantoin	0	0	1 (1) [2c]	0	0	**1 (1)**
	J01XX04 Spectinomycin	J01MA02 Ciprofloxacin	J01AA02 Doxycycline	P01AB01 Metronidazole	0	0	0	1 [4]	0	**1**
	J01FA10 Azithromycin	J01MA01 Ofloxacin	J01MA02 Ciprofloxacin	J01AA02 Doxycycline	1 [4]	0	0	0	0	**1**

Four antibiotics, annual number of cases					**1**	**0**	**1 (1)**	**1**	**0**	**3 (1)**

**Table:** (b) Female cases (*n* = 15)

	ATC code of antibiotic 1/antibiotic substance 1	ATC code of antibiotic 2/antibiotic substance 2	ATC code of antibiotic 3/antibiotic substance 3	ATC code of antibiotic 4/antibiotic substance 4	2009	2010	2011	2012	2013	Total
Total number of female cases recorded					**1**	**2**	**2**	**5 ** **(1)**	**5 ** **((1))**	**15** ** (1)** ** ((1))**

No antibiotic recorded					**0**	**1** **[2]**	**0**	**2** **[2, 3]**	**1** **[2]**	**4**

One antibiotic recorded	J01DD04 Ceftriaxone				0	1 [4]	1 [3]	1 [2]	1 [4]	**4**
	J01CA04 Amoxicillin				1 [4]	0	1 [4]	1 [2]	0	**3**

Number of cases, one antibiotic recorded					**1**	**1**	**2**	**2**	**1**	**7**

Two antibiotics recorded	J01DD04 Ceftriaxone	J01FA10 Azithromycin			0	0	0	0	1 [4]	**1**
	J01FA10 Azithromycin	P01AB01 Metronidazole			0	0	0	0	1 ((1)) [3t]	**1 ((1))**

Number of two antibiotics recorded					**0**	**0**	**0**	**0**	**2 ((1))**	**2 ((1))**

Three antibiotics recorded	J01DD04 Ceftriaxone	J01FA10 Azithromycin	J01XE01 Nitrofurantoin		**0**	**0**	**0**	**1 (1)** **[2c]**	**1** **[3]**	**2 (1)**

**Table 4:** Prescription of ceftriaxone, spectinomycin, and/or azithromycin for gonorrhoea cases registered in Flemish general practice 2009–2013 (*n* = 91), Intego database; in brackets (): number of cases with chlamydia coinfection; in double brackets (()): number of cases with genital trichomonas coinfection.

	2009	2009 (%)	2010	2010 (%)	2011	2011 (%)	2012	2012 (%)	2013	2013 (%)	Total
Total number of gonorrhoea cases recorded	15 (1)		22 (2)		17 (4)		21 (3)		16 ((1))		91 (10) ((1))

No antibiotic recorded	1	7%	5	23%	0	0%	4	19%	3	19%	13

Prescribed antibiotic(s) include											
Ceftriaxone	0	0%	3	14%	6 (2)	35%	6 (2)	29%	9	56%	24 (4)
Spectinomycin	2 (1)	13%	1	5%	2 (1)	12%	4	19%	0	0%	9 (2)
Ceftriaxone and spectinomycin	0	0%	0	0%	1 (1)	6%	1	5%	0	0%	2 (1)
Ceftriaxone and/or spectinomycin	2 (1)	13%	4	18%	7 (2)	41%	9 (2)	43%	9	56%	31 (5)
Azithromycin	2	13%	2	9%	6 (3)	35%	7 (3)	33%	10 ((1))	63%	27 (6) ((1))
Ceftriaxone and/or spectinomycin together with azithromycin	0	0%	1	5%	3 (2)	18%	4 (2)	19%	7	44%	15 (4)
