# Supplementary material for: Prescription of Antibiotics to Treat Gonorrhoea in General Practice in Flanders 2009–2013: A Registry-Based Retrospective Cohort Study
Source: J Sex Transm Dis. 2017 Jul 31;2017:1860542. doi: 10.1155/2017/1860542 (PMC5555009; doi:10.1155/2017/1860542)
Supplement: Supplementary file 1 — The Supplementary Material contains (1) the selected Medidoc codes, the associated diagnostic group code, the Medidoc clinical label in Dutch and its English equivalent; (2) ATC codes for the selection of antiinfectives from the Intego database. [file 1860542.f1.doc]

Supplementary material

*Annex 1*

Chlamydia, gonorrhoea, syphilis, and trichomonas diagnostic group codes, Medidoc codes, and Medidoc clinical label in Dutch selected for extraction, the latter with English equivalent

| **Diagnostic group code (English equivalent)** | **Medidoc internal code** | **Medidoc clinical label (keyword)** | **English equivalent of Medidoc clinical label** |
| --- | --- | --- | --- |
|  |  |  |  |
| **CHLAMYDIA** |  |  |  |
| CHLAMYDIA | AA5610 | Endometritis door Chlamydia | Chlamydial endometritis |
| CHLAMYDIA | AA5611 | Salpingitis door chlamydia | Chlamydial salpingitis |
| CHLAMYDIA | AA5613 | Bekkenontsteking door chlamydia | Chlamydial pelvic inflammation |
| CHLAMYDIA | AA569 | chlamydia s.o.a. | Chlamydial STD (sexually transmitted disease) |
| CHLAMYDIA | DA563 | Rectitis chlamydia | Chlamydial inflammation of the rectum |
| CHLAMYDIA | MDC_X92 | Chlamydia-infectie geslachtsorg[aan] vrouw | Chlamydial infection of female sexual organ |
| CHLAMYDIA | UA5601 | Chlamydia cystitis | Chlamydial cystitis |
| CHLAMYDIA | UA5602 | Chlamydia urethritis | Chlamydial urethritis |
| CHLAMYDIA | UA562 | Chlamydia urogenitaal | Urogenital chlamydia |
| CHLAMYDIA | X61601 | Cervicitis chlamydia | Chlamydial cervicitis |
| CHLAMYDIA | X61613 | Vulvovaginitis chlamydia | Chlamydial vulvovaginitis |
| CHLAMYDIA | XA5600 | Chlamydia cervicitis | Chlamydial cervicitis |
| CHLAMYDIA | XA5603 | Chlamydia vulvovaginitis | Chlamydial vulvovaginitis |
| CHLAMYDIA | YN513 | Epididymitis door chlamydia | Chlamydial epididymitis |
|  |  |  |  |
| **GONORRHOEA** |  |  |  |
| GONORRHOEA | AA549 | Gonorroe | Gonorrhoea |
| GONORRHOEA | AA5491 | Gonorroe | Gonorrhoea |
| GONORRHOEA | AA5492 | Infectie door Neisseria gonorrhoeae | Neisseria gonorrhoeae infection |
| GONORRHOEA | AA5494 | Gonorroe | Gonorrhoea |
| GONORRHOEA | AA5498 | Gonorroe | Gonorrhoea |
| GONORRHOEA | AA5499 | Gonorroe | Gonorrhoea |
| GONORRHOEA | B09889 | Gonokokkemie | Gonococcaemia |
| GONORRHOEA | DA546 | Gonokokken rectitis | Gonococcal proctitis |
| GONORRHOEA | DA5461 | Gonokokken rectitis | Gonococcal proctitis |
| GONORRHOEA | HA545 | Farynx infectie door gonokokken | Gonococcal pharynx infection |
| GONORRHOEA | KI3201 | Pericarditis akuut infektieus bacterieel gonokokkaal | Acute infectious bacterial gonococcal pericarditis |
| GONORRHOEA | KI3981 | Endocarditis gonokokken | Gonococcal endocarditis |
| GONORRHOEA | KI4102 | Myocarditis gonokokken | Gonococcal myocarditis |
| GONORRHOEA | L0985 | Artritis gonorroeisch | Gonorrhoeal arthritis |
| GONORRHOEA | LM730 | Bursitis door gonokokken | Gonococcal bursitis |
| GONORRHOEA | MDC_X71 | Gonorroe vrouw | Female gonorrhoea |
| GONORRHOEA | MDC_Y71 | Gonorroe man | Male gonorrhoea |
| GONORRHOEA | U0980 | Infektie gonokokken urethra akuut | Acute gonococcal urethra infection |
| GONORRHOEA | U09801 | Urethritis gonorroe akuut | Acute gonorrhoeal urethritis |
| GONORRHOEA | U0982 | Infektie gonokokken urethra chronisch | Chronic gonococcal urethra infection |
| GONORRHOEA | U583811 | Nefritis interstitieel + gonokokken infektie | Interstitial nephritis and gonococcal infection |
| GONORRHOEA | UA5403 | Gonokokken urethritis | Gonococcal urethritis |
| GONORRHOEA | UA54031 | Urethritis gonokokken | Gonococcal urethritis |
| GONORRHOEA | UA54039 | Gonorroe urethritis | Gonorrhoeal urethritis |
| GONORRHOEA | UN2911 | Gonokokken ureteritis | Gonococcal ureteritis |
| GONORRHOEA | UY5403 | Urethritis door gonokokken Y | Male gonococcal urethritis |
| GONORRHOEA | UY54031 | Urethritis door gonokokken Y | Male gonococcal urethritis |
| GONORRHOEA | W6471 | Zwangerzap en gonorroe | Pregnancy and gonorrhoea |
| GONORRHOEA | X098 | Infektie gonokokken | Gonococcal infection |
| GONORRHOEA | X0980 | Infektie gonokokken acuut X NNO | Acute female gonococcal infection, not further specified |
| GONORRHOEA | X09800 | Bartholinitis gonokokken acuut | Acute gonococcal bartholinitis |
| GONORRHOEA | X09802 | Vulvovaginitis gonokokken acuut | Acute gonococcal vulvovaginitis |
| GONORRHOEA | X09815 | Cervicitis acuut gonokokken | Acute gonococcal cervicitis |
| GONORRHOEA | X09816 | Endometritis acuut gonokokken | Acute gonococcal endometritis |
| GONORRHOEA | X09817 | Salpingitis acuut gonokokken | Acute gonococcal salpingitis |
| GONORRHOEA | X09821 | Bartholinitis gonokokken chronisch | Chronic gonococcal bartholinitis |
| GONORRHOEA | X09823 | Vulvovaginitis gonokokken chronisch | Chronic gonococcal vulvovaginitis |
| GONORRHOEA | X09835 | Cervicitis gonokokken chronisch | Chronic gonococcal cervicitis |
| GONORRHOEA | X09836 | Endometritis gonokokken chronisch | Chronic gonococcal endometritis |
| GONORRHOEA | X09837 | Salpingitis gonokokken chronisch | Chronic gonococcal salpingitis |
| GONORRHOEA | X09839 | Gonorroe chronisch X NNO | Chronic female gonorrhoea, not further specified |
| GONORRHOEA | X61614 | Vulvovaginitis gonococcen [sic] | Gonococcal vulvovaginitis |
| GONORRHOEA | XA54011 | Cervicitis gonokokken | Gonococcal cervicitis |
| GONORRHOEA | Y09812 | Infektie gonokokken acuut prostaat | Acute gonococcal prostate infection |
| GONORRHOEA | Y09813 | Infektie gonokokken acuut testis | Acute gonococcal testis infection |
| GONORRHOEA | Y09814 | Infektie gonokokken acuut zaadblaasjes | Acute gonococcal infection of the seminal vesicles |
| GONORRHOEA | Y09819 | Infektie gonokokken acuut Y NNO | Acute male gonococcal infection, not further specified |
| GONORRHOEA | Y0982 | Infektie gonokokken chronisch | Chronic gonococcal infection |
| GONORRHOEA | Y09832 | Infektie gonokokken chronisch prostaat | Chronic gonococcal prostate infection |
| GONORRHOEA | Y09833 | Infektie gonokokken chronisch testis | Chronic gonococcal testis infection |
| GONORRHOEA | Y09834 | Infektie gonokokken chronisch zaadblaasjes | Chronic gonococcal infection of seminal vesicles |
| GONORRHOEA | Y09838 | Infektie gonokokken chronisch rectum Y | Chronic male gonococcal rectal infection |
| GONORRHOEA | Y09839 | Infektie gonokokken chronisch Y NNO | Chronic male gonococcal infection, not further specified |
|  |  |  |  |
| **SYPHILIS** |  |  |  |
| SYPHILIS | A0907 | Syfilis congenitaal laat NNO | Late congenital syphilis, not further specified |
| SYPHILIS | A0909 | Syfilis congenitaal NNO | Congenital syphilis, not further specified |
| SYPHILIS | A0910 | Syfilis primair genitaal | Primary genital syphilis |
| SYPHILIS | A09130 | Syfilis secundair + exantheem | Secondary syphilis and exanthema |
| SYPHILIS | A0958 | Syfilis tertiair | Tertiary syphilis |
| SYPHILIS | A096 | Syfilis latent laat | Latent late syphilis |
| SYPHILIS | A0971 | Syfilis serologie positief | Positive syphilis serology |
| SYPHILIS | A0979 | Syfilis NNO | Syphilis, not further specified |
| SYPHILIS | AA5103 | Primaire genitale syfilis | Primary genital syphilis |
| SYPHILIS | AA5112 | Primaire anale syfilis | Primary anal syphilis |
| SYPHILIS | AA5121 | Primair extragenitaal syfilis X | Primary extragenital female syphilis |
| SYPHILIS | AA528 | Syfilis laat latent | Late latent syphilis |
| SYPHILIS | AA5282 | Syfilis laat latent | Late latent syphilis |
| SYPHILIS | AA5292 | Syfilis laat | Late syphilis |
| SYPHILIS | AA530 | Syfilis latent NNO | Latent syphilis, not further specified |
| SYPHILIS | AA5391 | Syfilis | Syphilis |
| SYPHILIS | AA5392 | Syfilis | Syphilis |
| SYPHILIS | AA65 | Endemische syfilis | Endemic syphilis |
| SYPHILIS | BA51492 | Secundaire syfilis | Secondary syphilis |
| SYPHILIS | K0930 | Aneurysma aortae syfilitisch | Syphilitic aortic aneurysm |
| SYPHILIS | K0931 | Aortitis syfilitisch | Syphilitic aortitis |
| SYPHILIS | K0932 | Endocarditis syfilitisch | Syphilitic endocarditis |
| SYPHILIS | K09381 | Pericarditis syfilitisch | Syphilitic pericarditis |
| SYPHILIS | K09382 | Myocarditis syfilitisch | Syphilitic myocarditis |
| SYPHILIS | KI3983 | Endocarditis door syfilis | Syphilitic endocarditis |
| SYPHILIS | KI4104 | Myocarditits door syfilis | Syphilitic myocarditis |
| SYPHILIS | L09001 | Pijn arm NNO [observations to be dropped due to internal Medidoc assignment error] | Arm pain, not further specified |
| SYPHILIS | L09001 | Syfilis congenitaal locomotorisch | Congenital locomotor syphilis |
| SYPHILIS | L09161 | Syfilis secundair bot | Secondary bone syphilis |
| SYPHILIS | MDC_X70 | Syphilis vrouw | Female syphilis |
| SYPHILIS | MDC_Y70 | Syphilis man | Male syphilis |
| SYPHILIS | N09490 | Syphilis gumma CZS | Syphilis gumma central nervous system |
| SYPHILIS | N09499 | Syfilis neurologisch NNO | Neurologic syphilis, not further specified |
| SYPHILIS | W6470 | Zwangerschap en syfilis | Pregnancy and syphilis |
| SYPHILIS | X0910 | Syfilis primair genitaal X | Primary genital female syphilis |
| SYPHILIS | X0911 | Syfilis primair anaal X | Primary anal female syphilis |
| SYPHILIS | X0913 | Syphilis secundair cutaan | Secondary skin syphilis |
| SYPHILIS | X0919 | Syfilis X NNO | Female syphilis, not further specified |
| SYPHILIS | X092 | Syfilis latent X | Latent female syphilis |
| SYPHILIS | X0958 | Syfilis laattijdig tertiair X | Late tertiary female syphilis |
| SYPHILIS | X096 | Syfilis laattijdig latent X | Late latent female syphilis |
| SYPHILIS | X61652 | Ulcus vulva syfilis | Syphilitic vulvar ulcer |
| SYPHILIS | Y0910 | Syfilis sjanker Y | Male syphilitic chancre |
| SYPHILIS | Y092 | Syfilis latent Y | Latent male syphilis |
| SYPHILIS | YA513 | Syfilis secundair mucocutaan Y | Secondary mucocutaneous male syphilis |
|  |  |  |  |
| **TRICHOMONAS** |  |  |  |
| TRICHOMONAS | A1319 | Infektie trichomonas NNO | Trichomonas infection, not further specified |
| TRICHOMONAS | AA599 | Trichomonas infectie | Trichomonas infection |
| TRICHOMONAS | MDC_X73 | Genitale trichomoniasis vrouw | Genital female trichomoniasis |
| TRICHOMONAS | U13102 | Urethritis trichomonas | Trichomonas urethritis |
| TRICHOMONAS | UN3701 | Trichomonas urethritis | Trichomonas urethritis |
| TRICHOMONAS | X13100 | Trichomonas fluor | Trichomonas fluor |
| TRICHOMONAS | X13101 | Vulvovaginitis trichomonas | Trichomonas vulvovaginitis |
| TRICHOMONAS | X13104 | Cervicitis trichomonas | Trichomonas cervicitis |
| TRICHOMONAS | XA5900 | Trichomonas vulvovaginitis | Trichomonas vulvovaginitis |
| TRICHOMONAS | Y13103 | Trichomonas prostatitis | Trichomonas prostatitis |

*Annex 2*

ATC groups of antiinfectives selected for extraction from the Intego database; antiinfectives with ATC codes beginning with D (dermatological antiinfectives) and S (sensory antiinfectives) were later excluded from the analysis, antiinfectives beginning with A, G, L, and R were not observed

| **ATC code** | **ATC group** |
| --- | --- |
| A01AB | Antiinfectives and antiseptics for local oral treatment |
| A07A | Intestinal antiinfectives |
| D06 | Antibiotics and chemotherapeutics for dermatological use |
| D07C | Corticosteroids, combinations with antibiotics |
| D09AA | Medicated dressings with antiinfectives |
| G01 | Gynecological antiinfectives and antiseptics |
| J01A | Tetracyclines |
| J01B | Amphenicols |
| J01C | Beta-lactam antibacterials, penicillins |
| J01D | Other beta-lactam antibacterials |
| J01E | Sulfonamides and trimethoprim |
| J01F | Macrolides, lincosamides and streptogramins |
| J01G | Aminoglycoside antibacterials |
| J01M | Quinolone antibacterials |
| J01R | Combinations of antibacterials |
| J01X | Other antibacterials |
| J05AA | Thiosemicarbazones |
| J05AB | Nucleosides and nucleotides excl. reverse transcriptase inhibitors |
| J05AC | Cyclic amines |
| J05AD | Phosphonic acid derivatives |
| J05AE | Protease inhibitors |
| J05AF | Nucleoside and nucleotide reverse transcriptase inhibitors |
| J05AG | Non-nucleoside reverse transcriptase inhibitors |
| J05AH | Neuraminidase inhibitors |
| J05AR | Antivirals for treatment of HIV infections, combinations |
| J05AX | Other antivirals |
| L01CB | Podophyllotoxin derivatives |
| P01A | Agents against amoebiasis and other protozoal diseases |
| R02AA | Throat preparations: Antiseptics |
| R02AB | Throat preparations: Antibiotics |
| R05X | Respiratory system, cough and cold preparations: other cold preparations |
| S01A | Ophthalmologicals: antiinfectives |
| S01C | Ophthalmologicals: antiinflammatory agents and antiinfectives in combination |
| S02A | Otologicals: antiinfectives |
| S02C | Otologicals: corticosteroids and antiinfectives in combination |
| S03A | Ophthalmological and otological preparations: antiinfectives |
| S03C | Ophthalmological and otological preparations: corticosteroids and antiinfectives in combination |
